# Negotiating filial care in transitions: an ethnographic study of family involvement in China’s nursing homes

**DOI:** 10.1186/s40711-023-00187-4

**Published:** 2023-05-15

**Authors:** Xinyue Wu

**Affiliations:** School of Humanities, Southeast University, No. 2 Dongnandaxue Road, Nanjing, China

**Keywords:** Aging, Eldercare, Filial piety, Institutional care, Family involvement

## Abstract

Based on ethnographic research conducted in two nursing homes in China, this article examines how institutional eldercare reshapes the expectations and practices of filial piety. It finds that families accept institutional care as a solution to the elderly care deficit. They expect a new division of care between labor and love, assigned to paid care workers and family members, respectively. This ideal of care division is rooted in the “intimate turn” in Chinese family life. Nevertheless, many family members go beyond this care division and remain deeply involved in nursing homes. On the one hand, adult children take on the responsibility to manage surrogate caretakers to improve the quality of care. On the other hand, they continue to provide personal care and companionship. Sharing family time is made the highest priority, especially in the face of impending death. This study goes beyond the binary division between commercial care and family care and sheds light on the transformation of filial piety in the commodification of eldercare in contemporary China.

## Introduction

China is facing an unprecedented rate of population aging. The Seventh National Population Census showed that there were 190.64 million citizens aged 65 and above, accounting for 13.50% of the total population. By 2050, nearly one-third of China’s population will be aged 60 or over.^1^ This demographic transformation will pose significant social challenges. Traditional norms of filial piety have long been considered the major cultural force holding together the system of familial eldercare. In the traditional norms, adult children should be entirely responsible for providing care and other support for their elderly parents. Until now, home-based care has been advocated as the predominant model according to the government’s 9073 plan,^2^ emphasizing family members’ care duties to elderly people.

However, accelerating urbanization and internal migration are eroding traditional models of family caregiving. Multigenerational co-residence is declining. Data from the national census show that the average number of people living in one household in China dropped from 3.96 in 1990 to 2.62 in 2020.^3^ Adult children, the expected future caregivers, become less available because of work pressures, a lack of time, and geographical distance. From a multiple-generational perspective, intergenerational competition for care resources also results in care deficits for the elderly (Zhong and Peng 2022).

Currently, various patterns of eldercare services are emerging to provide alternatives to family care. For many middle-class families, hiring domestic workers provides a means to meet their care demands. The Chinese government has also introduced a series of policies to promote the eldercare service industry as a supplement to family care, leading to the rapid development of institutional care. By the end of 2020, China had a total of 38,000 registered eldercare institutions, up 10.4% from the previous year. The latest national five-year plan sets a target of 9 million total eldercare beds, with 55% serving the disabled, elderly community. According to traditional filial norms, placing parents in an eldercare institution has long carried the stigma of being unfilial, resulting in a loss of face for both parents and their adult children (Zhang 2016). Nevertheless, an increasing number of urban families consider institutional care a viable means of providing professional long-term care for their frail elderly parents, particularly in metropolitan cities such as Beijing and Shanghai (Liu 2021:133).

The expansion of institutional care breaks down the wall between market and family. Notably, I have found that familial and commercial care are combined, and family members and paid care workers collaborate to provide eldercare. The new realities not only call for a remaking of the moral vision of eldercare but also pose challenges to families, such as reinterpreting the meaning of filial piety and negotiating and realizing good care in an institutional setting. Keeping the focus on families’ roles in nursing homes, this article explores the transformation of filial piety and sheds light on the intersections of the public and private spheres, economy, and intimacy in the age of outsourcing.

### Literature review: filial piety in the age of outsourcing

The purchase of commodified services has become a way for people to express love, maintain intimate relationships, and fulfill obligations. This includes care, which used to be associated with households and the private realm but has now entered the market sphere (Hochschild 2003; Zelizer 2005; Constable 2009). Specialist services for children and elderly care are increasingly being subcontracted to domestic workers and care facility staff. However, the integration of economic activities into the realm of intimacy often raises moral concerns and resistance. Some believe that the economy and intimacy are separate and incompatible worlds, with one based on calculation and efficiency and the other based on sentiment and affective bonds. It is feared that mixing the two spheres could result in disorder and moral decay.

Scholars have contributed to the debate on the relationship between intimacy and economy. In contrast to the “hostile worldview” mentioned above, Zelizer (2005) offered an analysis focusing on “connected lives” and how people navigate the complex intersection of economic activity and intimacy. It is especially important as paid care increasingly enters the realm of the family, leading to social and cultural tensions. As a result, boundary-making in outsourcing care has become a crucial issue. Many studies have examined employer-employee relationships, shedding light not only on the gender, class, and racialized boundaries that exist in everyday life but also on broader social inequalities (Lan 2006; Boris and Parreñas 2010).

Care outsourcing may also redefine how we express love and devotion to family. For example, Lan and McDonald’s work describes how mother-employers create and monitor a boundary between the sacred realm of the maternal and the more mundane tasks of the caregiver (Lan 2006; McDonald 2011). Hochschild (2013) suggests that some important questions need to be answered: at what points do we locate the notion of the sacred? In other words, what aspects of care do we consider too important to be outsourced? How will care outsourcing impact the “authenticity” of our intimacy?

When we discuss eldercare in China, I argue that the notion of filial piety places a moral boundary between the market and family, directing the practices and feelings of those involved in the process of market transfer. Filial piety, a cornerstone of Chinese Confucian ethics, calls on adult children to fulfill moral obligations to respect, obey, support, and serve their elderly parents. This set of moral codes has shaped intergenerational relationships and behavior for centuries (Ikels 2004; Shea Moore and Zhang 2020). Filial piety has long been intertwined with the family-centered care regime, where adult children are responsible for providing elderly care to their parents. It has also established a moral evaluation system that defines some acts as filial, such as taking care of one’s elderly parents in daily life and making them happy, and others as unfilial, such as neglecting or dishonoring them. Therefore, filial piety directs a set of “feeling rules” that govern how we feel across a range of situations (Hochschild 2003). For example, adult children may feel shame and guilt if they violate some codes of filial piety. Such feeling rules are deeply internalized and play an essential role in moral self-making.

Filial piety remains an important ideological concept that shapes an individual’s moral values and behavior. However, recent research has revealed that the understanding of filial piety changes over time due to significant demographic, economic, and social changes (Ikels 2004; Yan 2003,2016; Zhang 2017; Shea et al. 2020). Tensions arise between traditional ideas and present realities and between moral rules and everyday ethics (Kleinman et al. 2011). A growing number of studies focus on changes in intergenerational relationships and the new practices and interpretations of filial piety. For example, Yan (2016, 2018) claimed that family sources and attention are focused downward on the youngest generation rather than on elderly individuals, paving the way for intergenerational intimacy. Other scholars have explored the increased flexibility of eldercare arrangements. It has been noted that a new eldercare industry, including residential care and in-home care services, has given rise to “a transfer chain of filial care” (Lan 2002) or a “commodification” of filial piety (Zhang 2017).

Although China is witnessing a growing degree of eldercare outsourcing, there is still a “public‒private” divide in current care studies (Xiao and Jian 2020): studies on family eldercare focus on negotiations and practices of filial piety within the home and care distributions among family members, while studies on commercial eldercare primarily focus on the labor process and the dyadic relationships between caregivers and care recipients. In particular, nursing homes have long been described as total institutions (Goffman 1961) and places of radical rupture (Gubrium 1997), while less attention has been given to the continuous family ties in these places. This article attempts to go beyond such a binary division and shed light on the complex interplays of economy and intimacy, family care and commercial care. It explores the following questions: How do families reconcile the moral norm of filial piety with the real need to seek institutional care? How does the commodification of eldercare reshape the expectations and practices of filial piety? How can an individual continue to be a filial son/daughter when he or she has outsourced care tasks to a stranger?

## Methodology

This article builds on ethnographic fieldwork conducted in two nursing homes in Yong’an (pseudonymous) between 2016 and 2021. Yong’an is a county-level city in Eastern China with more than 350,000 citizens over the age of 60, accounting for 21% of the permanent population. With the increase in the number of aged persons with disabilities and dementia, domestic care services and residential care facilities are common choices for an increasing number of urban middle-class families. Currently, the city has more than 20 nursing homes, and the number is growing.

Yong’an was selected as the research site for two reasons. First, Yong’an is located in the Yangtze River Delta region, one of the wealthiest as well as the earliest demographically aging regions in China; thus, the local government has been encouraging private actors to participate in the senior care industry in recent years, resulting in notable changes in the landscape of eldercare. Second, as a county-level city, Yong’an should be understood as a hybrid of urban and rural culture, implying the presence of dynamics between continuity, transition, and overlapping modern and traditional values. It also provides a unique perspective on China’s grassroots society and its operations. For local eldercare institutions, promoting the marketization and professionalization of care services has become a theme, but everyday care practices are still largely guided by life experiences and ordinary ethics.

I conducted my fieldwork in two nursing homes: Healthcare Homeland was established more than ten years ago and has more than 370 residents, and Sincerity was established only two years ago and has approximately 150 residents. Few are independent among the residents, and most have various degrees of physical and cognitive disability. The cost ranges from 2000 to 4000 yuan per month, depending on the room type and the level of care. Most senior residents have a retirement pension and can cover the fee by themselves. A few residents with rural hukou receive lower pension payments and need financial support from their children. I chose two nursing homes to explore whether institutional management would affect the culture of care and family involvement, but I found more similarities between them. Both nursing homes lack professional management and training, and the quality of care is generally not high enough, only meeting daily living needs. Everyday care services rely mainly on a group of middle-aged and older women from nearby rural areas. These care workers are mostly from marginalized backgrounds with low levels of education and insufficient professional training. I mostly worked with them, dressed in a white uniform as they did. The ethnographic data relied heavily on participant observation and open-ended interviews with institutional staff, residents, and visiting relatives. Afterward, I conducted semi-structured interviews with family members in their homes to allow them to speak more freely about their attitudes and complex feelings about institutional care.

### Market transfer and a new understanding of filial care

For a long time, China’s social welfare institutions have accommodated elderly people with no children, no income, and no relatives, who are termed the “three nos.” The traditional Confucian ethical system stresses core values of family integrity and continuity, which generate life meaning for every individual. Elderly individuals without descendants or family support who are placed in institutions are considered to have the most unfortunate fate. Social welfare institutions have long been imagined as “zones of social abandonment.” Placing parents in a nursing home is considered to be a violation of filial norms.

Although eldercare institutions have proliferated in recent years, primarily in urban areas, this stigma has far-reaching effects. Elderly individuals are rarely willing to accept institutional care. According to the 2014 China Longitudinal Aging Social Survey, only 3.73% of seniors chose institutional care, while most preferred to live in their own homes or their children’s homes and have family members take care of them (Du et al. 2016). Sending parents for care in institutions also puts adult children in an ethical dilemma. They may feel guilty and face public embarrassment. Therefore, under what circumstances do adult children or their parents decide to accept institutional care? How do they justify their decisions? What are their new expectations of filial care in institutional settings?

#### Family care deficit

China’s life expectancy increased to 77.93 years in 2022. Elderly people live longer and are in potential need of care for longer than ever before. In general, spouses are the most common primary caregivers of seniors. With increasing age, elderly individuals are more likely to experience widowhood and depend more on their adult children for direct care (Du et al. 2016). Elderly parents above 80 years of age rely mainly on their adult children and spouses for daily care, most of whom are in their 60 s and 70 s and may have difficulties meeting care demands (Chen and Shi 2020). Furthermore, from a four-generational perspective, those in their 60 s or 70 s also play a central role in caring for their grandchildren. As families give priority to children, the youngest generation will likely crowd out family care resources for elderly members (Zhong and Peng 2022). Seniors over 80 in poor health with disabilities or dementia are particularly vulnerable to family care deficits. This is the portrait of residents in nursing homes today.

Entering a nursing home implies a “biographical disruption” (Bury 1982) in which routine home-based life is threatened by weakness and dependency in advanced age. Most residents and their children I interviewed expressed mixed feelings about the decision, and some children said they felt guilty and upset about not being able to take care of their parents by themselves. One daughter told me that she even cried the night before sending her mother to a nursing home. Making such a care decision could be regarded as a “moral breakdown,” which means an ethical moment when some event intrudes into everyday life and forces someone to consciously reflect upon the appropriate ethical response (Zigon 2009). The following section analyzes the narrative logic of senior residents and their children to articulate the moral reasoning process informed by personal life experiences and filial norms.

Man Jun used to live with her 80-year-old mother. As her mother’s health deteriorated, Man Jun eventually placed her mother in a nursing home. She said the following:

“We can’t handle it at home anymore. I have to work, and my sister is busy too. I think residential care is a future direction. After all, it is professional, and everyone is monitored. We do feel uneasy employing a maid to look after her at home. My mom is in poor health and has been hospitalized many times. Once when I came home from work, I found her collapsed on the floor with high blood sugar levels. It was very dangerous. We have to leave at seven in the morning and return at five in the evening. She was home alone, and no one knew that she had fallen. Safety is our main concern; otherwise, we wouldn’t have been willing to send her there. I hope she will live longer and lead a livelier life there. I also promised her that I would visit her often and pick her up on holidays.”

Man Jun’s story reveals a common family care crisis experienced when elderly parents begin to have difficulty maintaining activities of daily living and adult children are too busy to provide full-time care, causing them to experience burnout and helplessness. In this case, institutional care was considered a better option, equipped with trained medical staff and care workers to provide 24-h intensive care. Man Jun emphasized that her intention was to reduce her mother’s falling risk and enable her mother to live longer. Indeed, in my interviews with family members, “Make them live longer in a nursing home” was said repeatedly. Thus, institutional care was repackaged as another way of practicing filial piety rather than as a form of abandonment or neglect (Zhang 2017).

From the perspective of the senior residents, “not to be a burden on the children” was a dominant narrative that justified their entry into eldercare institutions. For example, 83-year-old Feng Ying was a retired manager who decided to move into a nursing home with her husband one year ago. She told me:

“We live here because my older son settled in America, and the second one is in charge of a local company and is busy too. We pay for the service with our pension, and the staff takes care of us. I need to be considerate of my children. It’s not that they are unfilial. They face a lot of pressure at work, have their own families, and have to take care of their own children and grandchildren. Currently, raising a child is not easy. I have to understand their position.”

Feng Ying is used to repeating this explanation due to constant inquiries from relatives and friends who wonder if her son and daughter-in-law have treated her badly by having her move into a nursing home. Concerned about her children’s pressures and lack of time, she rejects the traditional notion of filial piety that children should be physically present and perform care duties to repay their parents’ devotion and sacrifices. Instead, she recognizes that family care resources are insufficient and should be allocated to the younger generation as a priority. Feng Ying’s claim reflects the script of neofamilism in contemporary China that the entire family should focus its resources, efforts, and care on the younger generation. Many scholars claim that child/grandchild-centered neofamilism poses potential ethical challenges to eldercare, leading to a care deficit for the elderly (Yan 2018; Zhong and Peng 2022). In Feng Ying’s case, we found that institutional care became a solution in times of family care crisis and was resignified as a sign of love and care for younger generations.

#### Labor and love: an ideal of care division

Based on the interviews, it has also been found that senior residents and their children adopt a cultural framework that divides love and labor to shape their perceptions of care outsourcing. This framework reflects their expectations for the roles of the family and the institutions and helps them navigate the moral breakdown brought by the care decision. Like Man Jun, adult children believe that outsourcing care to the market allows them to subcontract most of their daily caregiving responsibilities while still feeling obligated to visit and spend time with their parents to maintain continuity and family integrity. In other words, labor is outsourced, but love continues. For most parents, it is not essential for their adult children to provide daily care, but they still expect frequent visits and displays of attention and care. A resident told me, “If my sons and daughters are unavailable, they do not have to come. If they have time to come, they don’t have to stay long or bring me anything either. All I need is for them to sit here and talk to me for a quarter or half an hour.”

Institutional staff also agree with such a division of care. They believe that regular visits and emotional support from family members play a greater role in improving the residents' quality of life. As a nursing home director told me, “The happiness of seniors depends not only on our services but also on family factors. For example, if a resident is treated well by the family and her children visit daily, she will feel happier. If no one comes to see her, she will definitely feel lost and lonely.” Family members are always encouraged to visit and participate in festival events. For example, some nursing homes hold foot-washing ceremonies on Mother’s Day or for the Chong Yang Festival, inviting adult children to wash their parents’ feet to display respect and gratitude. Such activities were meant to promote filial piety and enhance the emotional bonds between parents and their children.

Most of the interviewees drew a line between ideas of “caring for” and “caring about”—the former emphasized hands-on care such as feeding, bathing, and toileting, while the latter emphasized feelings of responsibility and emotional attachment—which are considered two essential elements of the definition of care (Abel and Nelson 1990). Such a division of care has been described by many scholars, particularly in mothering and domestic work: menial tasks associated with dirt and disorder are usually assigned to paid workers, and homemakers tend to take over tasks involving intimate contact and spiritual housework. The division of care labor always interacts with racial/ethnic hierarchies in specific cultural and social contexts (Duffy 2005; Lan 2006).

Notably, there is a similar stratified division of filial care here, based on the split between labor and love. In this context, “love” refers to the intimate connection and expressions of affection and concern within the family. In contrast, “labor” refers to the ongoing efforts to sustain the daily life of care recipients, which entails repeating the same caregiving activities on a regular basis. Expressions of caring and love are highlighted as the most important ways of practicing filial piety, while the labor of day-to-day care is downplayed and could be transferred to an outsider. This differs from the traditional notion of filial piety, which prioritizes serving parents as a major aspect of enacting filial virtue. For example, in *The Twenty-four Cases of Filial Piety,* a classic text of Confucianism, there were stories about how a son tasted and served medicine to his mother and how another son fanned his mother’s pillow and warmed her blanket to help his mother sleep comfortably. How can we understand this transformation of filial duties and the current division of filial care in the market transfer process?

As many studies indicate, there has been an “intimate turn” in Chinese family life in recent decades. The intergenerational relationship is characterized by a high degree of mutual caring and appreciation, as well as increasing emotional communication (Zhong and Ho 2014; Yan 2016,2018). The 2014 China Longitudinal Aging Social Survey showed that 47.9% of seniors considered “expressions of caring and love” to be the most important means to enact filial piety, and only 22.1% thought it was most important to “take care of their parents in daily life.” The data also showed that seniors with urban hukou and more education placed more value on children’s expressions of caring and intergenerational emotional bonding than on the provision of daily care or financial support (Liu 2021:169–223). Most senior residents agreed that the younger generation should not undertake too much of a care burden but rather focus time and effort on pursuing their own success and happiness. In addition, they mentioned that parents and children living separately could remove a major source of family conflict and promote intergenerational harmony. From the perspectives of adult children, outsourcing care could relieve long-term stress and related negative emotions, leading to a greater sense of closeness. In short, the development of reciprocity and emotional intimacy between generations has impacted the redistribution of eldercare, allowing market subcontracting to gain moral justification.^4^

### Managing the filial surrogate

Thus far, people commonly distinguish between physical and emotional care in the market transfer of care duty. When staff manages most residents’ needs 24 h a day, family members are expected to visit and accompany the seniors regularly. However, due to their moral obligations and distrust of commercial care, many families take on the responsibility of managing surrogate caretakers as if they are extensions of the children themselves. Their strategies include monitoring and directing care services and establishing a  *renqing* (人情) network to ensure the quality of care.

#### “Careless” commercial care: fact and opinion

Although China’s eldercare sector is growing rapidly, it is confronting many challenges and structural limitations, such as financial strain and a lack of professional staff and standard of care practices. Whether public or private, most nursing homes are incapable of providing high-quality professional care. Current eldercare services rely mainly on a group of middle-aged and older women from marginalized backgrounds with low levels of education and little professional training, whose caregiving skills come mostly from past experience caring for family members (Wu 2021). In nursing homes in Yong’an, one caregiver provided services to five or six residents, and the ratio was even higher during the night shift. The organization of care within the nursing home turned the complex work of caretaking into a streamlined mechanical task, draining the energy needed to provide affectionate care. Heavy workloads, burnout, and the social stigma of doing “dirty work,” affect caregivers’ work attitudes and even lead to abuse and humiliation (Wu 2018).

Recently, news and videos of elder abuse in institutions and homes have spread widely on social media, increasing the public’s mistrust of stranger caretakers and eldercare institutions. For example, an alarmist story posed by a WeChat public account attracted widespread attention with more than 100,000  views. It was about an aged man without a partner or children: he had paid 4 million to move into a high-quality nursing home in Guangzhou but died of negligence and abuse. The article said, “This older person had no children, no family, and no visitors. The care workers figured out that he had no one to rely on and therefore humiliated and mistreated him. Finally, he died in utter desperation two months later.” Although the story was later proven to be a fabrication, it mirrored the moral panic surrounding commercialized eldercare in China today. It also emphasized the traditional filial norm that the elderly should be cared for by their children rather than living in a nursing home dependent on nonfamily caretakers.

The structural constraints of the eldercare service industry today, along with the stereotypes and stigmas attached to nursing homes, give rise to family members’ mistrust and anxiety. In other words, their worries and suspicions are not only from traces of inadequate care in reality but also from the long-standing view of “hostile worlds” based on the supposition of “a sharp division between the diffuse, sentimental, and noncommercial world of the family and the specialized, impersonal, and commercialized world of goods and services outside the family” (Zelizer 2005:172). One daughter told me, “It’s just a job for the staff, so how can they take care of the elderly like family members?” When nursing homes move a share of eldercare from households to professional settings, family members generally use their experiences as caregivers as a baseline for evaluating the commercialized care services provided. The latter might always be perceived as task-oriented, lacking emotional involvement and personal attention, and degrading residents to objects of care treated as a part of a workload. Feelings of mistrust and anxiety thus lead family members to take responsibility for safeguarding the quality of care.

#### Monitoring daily care

Family members adopt various strategies to monitor institutional care services and seek a sense of security and control. Since most family members are busy and can not be present very often, WeChat has become the most important tool to enable them to keep track of their parents’ well-being and monitor the quality of care provided. It creates a virtual space, bringing formal and informal caregivers together across physical distance. For example, family members regularly check with staff about medication use, blood pressure, and blood sugar of their parents or about whether they can get up and walk by themselves. Care worker supervisors and social workers also send photos and short videos of residents’ daily activities to families, recording them doing finger exercises, singing together, or playing Mahjong. Such selected photos and videos always present a warm and peaceful environment, conveying that the nursing home is operating well and providing compassionate care service, trying to reassure family members.

Regular visits are another means to oversee the quality of care. Adult children, especially those who live close by and have no other caregiving responsibilities, often come to accompany their parents while monitoring how their parents are treated in detail and checking on daily care conditions, such as personal hygiene standards, the cleanliness of the environment, and staff attitude. They also observe how care workers interact with other residents. The regular visits enable them to find problems and take steps to improve care quality. Sometimes they ask care workers to adapt their work approach, and sometimes they even provide personal care to ensure their parents’ dignity and comfort.

Family members often complain about the care provided as too impersonal. For example, they told me that staff always made residents wear incontinence underwear all day regardless of their level of continence. Some care workers even double-diapered residents so that they could only change smaller soiled inner pads and prevent bedwetting. It was uncomfortable and could cause a rash. Some family members expressed their concerns to the caregivers. They thought incontinence underwear was unnecessary for residents who still had the motor capacity to use the bathroom by themselves and could result in increased dependency and a loss of autonomy. In addition, some adult children told me that while their parents were in declining health and needed assistance to get dressed and out of bed, the care workers were busy with multiple caretaking tasks and often left them lying in bed for too long, potentially resulting in muscle loss, joint degeneration, and depression. Thus, the families often remind the staff to help their parents get up and do exercises to stay active. They also frequently ask the staff to take their parents outside to get some sunshine. In the eyes of the family members, good care is about meeting seniors’ physical needs and maintaining their autonomy and social participation. More often, I saw that adult children themselves came to walk with their parents, helping them practice their mobility.

Family members also teach staff how to deliver care and communicate with their parents in the right way, given their knowledge of their personalities and life experiences. One daughter described how her mother, who had severe dementia, frequently got angry and scolded her caregiver. Later, she realized that the caregiver always asked her mother, “Who am I, and who is this visitor?” Her mother was unable to answer these questions or remember the right names, which damaged her self-esteem. Sometimes, her mother was irritated by the caregiver’s constant demands to get up and walk. The daughter found that the caregiver was responsible but used the wrong mode of communication. Thus, she suggested that the caregiver use an encouraging, motivational approach rather than constantly pressing her mother to be more active.

#### Establishing a *renqing* (人情) network

Although many family members think the institutional care provided is not good enough, they still try to maintain good relationships with the staff. A *renqing* (人情) network in the nursing home enabled people to convert work relations to familial ties. As a system of ethics, *renqing* involves emotional attachment, moral obligation, and rational calculation, guiding and regulating the way people relate to each other (Yan 1996).

As Yong’an is a county-level city with intensive *guanxi* networks, family members can find personal connections through kinship, their neighborhood, or community with staff members, which play an important role in trust building. This might even be the main reason why the families chose this particular nursing home. Gift giving was a primary means by which family members continually maintained and cultivated *renqing* (人情) with the staff, especially those care workers providing daily care to their parents. One daughter told me that gifts to each of her mother’s caregivers included a box of fine fruit for the last Spring Festival, a box of mooncakes for the last Mid-Autumn Festival, and a mixed nut pack for the New Year, not to mention apples and oranges from time to time. Another daughter told me that when she brought fruits and snacks for her mother each time, she often prepared an extra share for the caregiver. She said, “I am grateful for their hard work, such as their night shifts. Although we pay for the service, it is a personal token of our appreciation.” However, she also complained about some caregivers’ rudeness and neglect and hoped the gifts would make them treat her mother more gently and with dignity.

In addition, two unique gifts are embedded in the Chinese cultural context. The first is red velvet banners with gold fringe and tassels emblazoned with Chinese phrases such as “caring for seniors with dedication and commitment.” This is a purely symbolic expression of thanks to the staff member. Each banner, and sometimes a photo recording of the family presenting the banner to the staff, was hung on the wall to draw public attention and showcase the high-quality services provided by the nursing home. The second is *hongbao*, a gift of money packed in a red envelope. Contrary to the public display of the red banner, *hongbao* is given to the caregiver secretly. It is much more like a form of bribery, usually offered by the family hoping to receive more comprehensive care services or making special requests for tasks such as bed changing services. However, some nursing homes have started to ban the acceptance of *hongbao* in consideration of their reputation, while banners are still encouraged.

Gift and commodity exchanges are inevitably interwoven in care institutions, especially those lacking professional regulations. These gift-giving activities have created an ongoing personal connection between the caregiver and the family. The gifts are not only an expression of appreciation and gratitude to the hard-working staff but also imply an instrumental intention to ensure the quality of care and protect vulnerable elders. Such gifts cause the receiver to feel a moral obligation to provide special attention and better care in return. Gift relations enable participants to assert moral personhood and resist the potential for dehumanizing relations in commodified care that reduce a person to an object (Buch 2014).

The *renqing* (人情) network has also expanded to other senior residents and their relatives. When children bring fruit and food to their parents, they often share with other residents. Once, I saw a daughter bring steamed pumpkin from home—one bowl for her mother and the other for the roommate. After serving her mother, she fed her mother’s roommate with severe dementia. During the fieldwork, I noticed she never discriminated against this person with dementia. Instead, she enjoyed shaking hands and greeting her every time, developing a unique mode of communication. Families develop caring and compassionate bonds with other residents over the course of their visits. Those who visit regularly are also familiar with each other. Sharing details of their own life stories and family affairs is another form of gift exchange that builds a sense of closeness. These connections allow them to care for each other’s elders, share caregiving experiences, and monitor care services, creating an informal collaborative care network.

### Doing filial piety in the nursing home

Although most of the daily physical care is transferred to the surrogate caretakers, many family members continue to provide personal care and companionship to their parents to maintain their identities and continuity of life. These are not only for practical purposes—to make up for inadequate institutional care and improve the residents’ quality of life—there are deeper moral sentiments behind them. Witnessing the vulnerability and slow physical decline of the elderly residents, family members feel a stronger moral imperative to enact filial piety and express their love in the residents’ last stage of life. At this point, copresence itself becomes a pure form of family solidarity and intergenerational intimacy.

#### Providing personal care

Life in a nursing home is often portrayed as tedious and frustrating, which partially matches my field observations. Most residents are caught between protection and restriction and between care and control. Some seniors gradually separate from their original communities and social ties when they move into nursing homes. They are not allowed to leave the facility freely unless they have a pass or are accompanied by their children. Nursing homes confine their daily activities to certain areas, including rooms, corridors, and courtyards, to reduce the chances of falling and injury. The nursing home routinely provided daily care and monitored residents’ physical health but paid less attention to their emotional and spiritual needs due to a lack of staff and knowledge. Residents were accustomed to sitting in silence for hours and waiting for three set meals each day, which became the most important events in the setting. They commonly described their state as “living a dead life” or “waiting to die” with a sense of self-deprecation and sadness, conveying the residents as stuck in sedentary life in the nursing home.

Many children have expressed concerns about their parents’ physical and cognitive decline in such an environment. They think prolonged social isolation and loneliness might increase seniors’ vulnerability, engendering hopelessness, depression, and a loss of self-worth. A daughter who often visited her mother, who had suffered a stroke, told me, “There’s no one to talk to. I’m worried that she’s going to lose her mind and not be able to communicate. She has been living here for almost six months while getting duller and weaker.” Another visiting child confirmed such worries: “Every time I come, I see them sit there in silence, no talking, no communication. It is too bad. So as a child, whatever you come here to do, even if only to stay for half an hour, is a comfort to her.” Due to the division of care between labor and love, as mentioned above, providing emotional support or daily communication is not considered an essential part of caregivers’ work but rather the families’ responsibility. Family members become residents’ connections to the outside world through regular visits and companionship. Greetings, small talk, and body touch, any opportunity to be cared for, revitalize the residents, helping them maintain a sense of continuity and reducing their loneliness.

When family members are too busy to visit, they often use communication technologies to maintain connections in and outside the nursing home. During the facility lockdowns of the COVID-19 pandemic, communication technologies became the only available media for intimacy and sociality. Some residents received phone calls from their children almost daily, routinely discussing their daily lives, including what they did and ate. These phone calls provide information about what was happening in their lives in the nursing home and help family members learn more about their emotional and physical health from their tone of voice. The phone calls also make seniors feel that they are regularly cared for.

However, the family’s role is more than that of “visitor” or “comforter.” Some adult children are more deeply engaged in everyday care and provide individualized care to their parents. I find that daughters (sometimes daughters-in-law) generally stay longer and provide more bodily care than sons. They often help their parents wash their hair, give them footbaths, clip their nails, and give them massages. Ya Min visited her 90-year-old mother every afternoon. Her caring and thoughtfulness impressed me immensely. She usually first prepared nutritional powders after arriving. As it was difficult for her mother to swallow tablets, Ya Min had to grind vitamin and calcium tablets into powder and dissolve them in water. The water was also mixed with honey to make it less bitter. After serving the nutrition supplement, Ya Min washed and massaged her mother’s feet to relieve swelling and gently rubbed in lotion. This physical contact was highly emotionally involved, creating a sense of closeness and intimacy between Ya Min and her mother. How adult children perform their care is influenced by traditional gender roles whereby women dedicate disproportionately more time than men to household care work, such as feeding, bathing, and cooking. As a result, women are generally ready to give physical touch or bodily care. They also have more knowledge of the materiality of the body. The “caring” and “thoughtfulness” of these daughters are embedded in long-standing gender ideologies and labor divisions.

Sometimes, the progress of the market transfer does not work smoothly when seniors have difficulties adapting to the new institutional environment. It requires the family to engage more deeply and share some daily tasks that the staff should have done. Thus, the division of care between the institution and the family is further blurred. For example, a social worker told me that many seniors initially felt a sense of loss and discontinuity, and some of them even cried and begged to go home. To help their parents adjust to the new environment, some adult children accompanied them and slept in the same room for almost a week. One lived with her mother, who had dementia, in the nursing home for eight days. In addition, her mother fiercely resisted her unfamiliar caregiver’s assistance in the shower, which is a common behavior in people with dementia. The staff could not handle her screaming, crying, and attacks during bath time due to a lack of specialized knowledge and skills. The daughter had no choice but to come to the nursing home every week to bathe her mother herself, which should have been part of the daily care services provided by the institutional staff. This case shows that care activities should be shared in various ways across the boundary between formal and informal care based on the specific condition of each senior resident.

#### Family time in the last stage of life

As most elderly residents have chronic diseases, dementia, and various degrees of disability, family members, staff, and seniors are aware that most of these residents will spend the last phase of their lives in a nursing home. More than one resident said they were prepared for the final day: “It’s not going to get better anyway. At our ages, we are inevitably getting closer to it (death).” For them, sharing “family time” with children and grandchildren is what they look forward to most. On the other hand, complex feelings of compassion, fear of loss, and a desire to repay their parents’ dedication drive family members to spend more time at the nursing homes. In this last stage of life, the “presence” of adult children becomes the most important way to express care and filial piety. Authentic caring and emotional intimacy are considered the core values of filial piety and could never be subcontracted.

Mr. Wang’s 84-year-old mother had severe dementia and lived in the Sincerity Nursing Home. He visited his mother three or four times a week and often accompanied her for walks outside to get sunshine. He also liked to take photos and short videos of his mother, recording the details of her daily life and the progress of her disease. Mr. Wang told me, “I stay for about an hour each time. Most conversations are pointless, such as repeatedly asking and answering the question, ‘Have you eaten yet?’ Her words are barely intelligible. However, we actually have more contact now than when she lived at home.” Mr. Wang told me his mother lived alone in her house before entering the nursing home. Wang only visited her on weekends when she could care for herself. However, now that his mother was dependent and had lost most memories, Wang accompanied her more often, sitting with her, talking to her, or watching TV together. Like Mr. Wang, many adult children expressed that they spent more time with their parents than before, creating a deeper sense of closeness and intimacy. Wang’s mother, although she could no longer remember the names of her children, looked happier and calmer with the family’s company.

The inevitable decline and mortality of the aging bodies indicate the residents’ vulnerability, eliciting ethical responses and affective intensity (Butler 2004). Most adult children are fully aware that dying and death are inescapable and that there are fewer opportunities to be with their parents. The shadow of death provokes their fear of loss and consciousness of the past, recalling early years of parental care and nurturing. Therefore, final years in the nursing home are fraught with moral weight, not only about fulfilling moral obligations to reciprocate but also about being a filial child and a good person.

Mei was recognized as a filial daughter by the staff and other residents. She brought homemade food to her mother almost every day. After having a massive stroke a year before, her mother was left paralyzed and unable to speak. Witnessing her mother's slow death, Mei said, “My mom is so weak now. There is no quality of life for her at all. She can eat today but may not be able to eat next time. I will not regret it later if I treat her well while she is alive.” Mei arrived at the same time each morning and stayed for many hours. In addition to feeding her mother, Mei sat by her mother, knitting sweaters. She could do no more than share the day with her fragile mother.

Mei often told me about her mother's hardships in raising her children, especially during long periods of poverty and deprivation. She still remembered years of food shortages when parents saved their limited food rations for their children. “My mum bore plenty of bitterness, especially in the years of communal dining halls. She had to raise us five children. Sometimes, she even ate carrot stalks, saving white rice for us. She was very skinny those years, as she always fed us first.” The food Mei brought implied compensation for her mother’s early experiences of deprivation and reciprocity for her nurturing. As a Chinese proverb says, “It’s common for a child to finally grow the desire to take care of his parents, only to realize that they are long gone.” It is thought to be the saddest feeling for adult children. The moral commitment to repay parental kindness entangled with uncertainty in the face of impending death drives family members to seize the last chance to provide care and comfort, enabling parents to enjoy life at this stage.

Like Mei, children who visit frequently also gain a reputation of filial piety at nursing homes. The staff and some residents often make moral evaluations of family members in private: someone who comes often and stays longer is labeled a filial child, and their parents are considered to have the fortune (*fuqi, 福气*); at the same time, those who rarely appear are thought to be less caring. I once heard a story from a group of care workers while on break. When an elderly man had a high fever and severe pain, the staff called his oldest daughter to pay a visit, yet the oldest daughter told them to call the younger one instead. The younger daughter said she was busy too. The care workers thought the elderly father was seen as a burden with no one who truly cared about him. It signified the moral failure of the family.

When parents and children understand that death is gradually approaching, the value of family integrity is always put first. Having a “family life” at its best at the end of one’s life has become a shared normative moral practice in contemporary China (Fang 2021). In the liminal space between living and dying, family members are expected to visit and perform mundane care activities—feeding, bathing, and massaging—or share a time without doing anything specific. These repetitive activities could be understood as routine rituals, enacting filial piety through hands-on body care and companionship rather than words. “Presence” itself is now given the utmost importance, and the materiality of the body becomes the central part of communication and interrelatedness. As Kleinman (2012) claims, presence is central to the giving of care, which means being there existentially, even when nothing practical can be done. It is also “a moral practice that makes caregivers, and at times even the care receivers, more present and thereby fully human” (Kleinman 2009: 293). In this sense, sharing family time plays a crucial role in realizing one’s moral self and signifying the moral integrity of the family.

## Conclusion

As China’s population grows older and traditional family-based eldercare diminishes, the commodification of care has become a major mechanism for adult children to arrange eldercare. The cultural meanings and social practices of filial piety are modified and transformed in the market transfer of care. First, a new understanding of filial care is emerging based on a division between labor and love: day-to-day technical care tasks can be subcontracted to care institutions and care workers, while emotional care is highly valued and should be provided by the family. This concept has been rooted in the “intimate turn” in Chinese family life in recent years. Second, field investigations reveal that family members go beyond this care division and remain deeply involved in nursing home activities. On the one hand, they manage surrogate caretakers to improve the quality of care. Adult children have tried to practice filial piety by proxy by monitoring and directing care services and establishing a *renqing* (人情) network. On the other hand, many family members continue to provide companionship and personal care by themselves. Especially when they realize that death is approaching, family time is put first. Therefore, the commodification of care does not indicate a weakening of intergenerational ties or the erosion of filial piety. Adult children utilize certain ethical tactics to maintain family integrity and create new connections and care practices in institutions. As a moral value, filial piety is still a dominant cultural norm imposed on social expectations and individual behavior. As an ordinary ethic, it is constantly transformed by human agency and social context.

This study also shows that family members, residents, and care workers constitute the foundation of caretaking. Many families involved in institutional care provided insights from their experiences into how to deliver individualized care and what improvements should be made. The facts provide an imagination of shared caring that requires constant collaboration between formal and informal caregivers. As Annemarie Mol stated, those who share care should “experiment, experience, and tinker together.” They must respect each other’s experiences, attend to everyone’s strengths and limitations, and engage in inventive, careful experiments to improve the mode of care and tackle new problems that arise (Mol 2008:56). In brief, it is necessary to break the public‒private and market–family dichotomy and foster a more inclusive care environment based on ongoing partnerships and practical ethics of care, which can help overcome the weaknesses of commercialized task-based eldercare.

## Data Availability

Not applicable.

## Footnotes

1 China Capable of Dealing with Problem of Aging Society, China Daily. https://global.chinadaily.com.cn/a/202011/03/WS5fa095c5a31024ad0ba82a45.html.

2 The ‘9073’ plan is a social care service plan for aged citizens. It represents a goal for 90% of seniors to acquire home-based care, 7% to obtain community nursing services, and 3% to rely on institutional care.

3 Average size of households in China 1990–2020, statista. https://www.statista.com/statistics/278697/average-size-of-households-in-china/..

4 This study merely presents a new cultural framework that has emerged in care outsourcing and offers a preliminary analysis of the social contexts that have contributed to its emergence. However, the current research is limited by a lack of field data on how actors negotiate the boundaries of love and labor, as well as the division of responsibilities between institutions and families in specific caregiving practices. Further research is needed to address these gaps. Additionally, the cultural framework presented here is based primarily on interviews with senior residents and family members who are regularly involved in care institutions. The perspectives of family members who are rarely present have yet to be explored and should be considered in future research.
